# Bone morphogenetic protein-7 regulates Snail signaling in carbon tetrachloride-induced fibrosis in the rat liver

**DOI:** 10.3892/etm.2012.720

**Published:** 2012-09-24

**Authors:** WAN-RONG BI, CAI-XIA JIN, GUO-TONG XU, CHANG-QING YANG

**Affiliations:** 1 Department of Gastroenterology and Digestive Disease Institute, Tongji Hospital, Tongi University School of Medicine, Shanghai 200090;; 2 Stem Cell Laboratory, Shanghai 200092;; 3 Department of Gastroenterology and Digestive Disease Institute, Tongji Hospital, Tongi University School of Medicine, Shanghai 200065, P.R. China

**Keywords:** bone morphogenetic protein-7, Snail, mesenchymal-epithelial transition, liver fibrosis

## Abstract

The aim of this study was to explore the molecular mechanism of the bone morphogenetic protein-7 (BMP-7) downregulation of Snail-mediated E-cadherin repression and mesenchymal-epithelial transition (MET) induction, since little is presently known about this issue. In this study, our aim was to elucidate the underlying mechanism by which cells acquire liver fibrosis characteristics after epithelial-mesenchymal transition (EMT). Cell cultures were exposed to Snail alone or in the presence of BMP-7; control cultures were exposed to medium only. The expression of the mRNA encoding α-smooth muscle actin (α-SMA), Snail and E-cadherin in rat liver epithelial cells was determined by real-time quantitative PCR (RT-PCR) and the main results were confirmed by ELISA. Cell differentiation was determined by analysis of the expression of α-SMA, Snail and E-cadherin by western blotting and co-immunoprecipitation. We demonstrated Snail-induced upregulation of mRNAs encoding α-SMA and downregulation of mRNAs encoding E-cadherin in rat liver epithelial cells when compared with unstimulated cells, and confirmed these results at the protein level. BMP-7 downregulated Snail-induced α-SMA and upregulated E-cadherin release compared with untreated and Snail-treated cells. In summary, we demonstrated that BMP-7 induces MET through decreased downregulation of Snail. In addition, Snail1 directly regulates Nanog promoter activity. Notch signaling is also involved in this process.

## Introduction

Epithelial-mesenchymal transition (EMT) is a critical developmental process that plays a central role in the formation and differentiation of multiple tissues and organs. During EMT, epithelial cells lose cell-cell adhesion and apical polarity and acquire mesenchymal features, including motility, invasiveness and resistance to apoptosis (1). One of the key hallmarks of EMT is loss of E-cadherin, a cell-adhesion protein that is regulated by multiple transcription factors, including Snail, Slug and Twist. These transcription factors act as E-box repressors and block E-cadherin transcription (2). Transforming growth factor (TGF)-β1 induces EMT in epithelial cells through the upregulation of Snail1 in Smad-dependent signaling (3). The inhibition of Snail1 in mesenchymal cells results in decreased Nanog promoter luciferase activity and loss of self-renewal characteristics *in vitro*. BMP-7 induces mesenchymal-epithelial transition (MET) through Snail1 and Nanog downregulation. In mesenchymal cells post-EMT, Snail1 directly regulates Nanog expression and loss of Snail1 causes liver fibrosis.

Snail1 and Snail2 belong to the Snail superfamily of zinc finger (ZF) transcription factors (3) and have emerged as important repressors of E-cadherin and inducers of EMT (4). Vertebrate Snail1 and Snail2 factors share a high degree of homology at the DNA-binding C-terminal region, containing four and five C2H2 ZFs, respectively, and at the N-terminal region that contains the SNAG transactivation domain (5). Snail1 and Snail2 present a similar modular organization of nuclear import sequences, distributed among several ZFs (6). Snail factors have emerged as essential regulators of physiological and pathological EMT processes (7). Post-translational modifications of mammalian Snail have been shown to modulate Snail1 stability and functional repressor activity. In particular, phosphorylation by GSK3β or PKD1 plays a negative role (8), while phosphorylation by PAK1, CK2, PKC or Lats2 or interaction with lysyl oxidase-like 2/3 (LOXL2/3), exerts a positive effect on Snai1 functionality (9).

Our hypothesis is that mesenchymal cells acquire liver fibrosis traits after EMT through Snail1-dependent mechanisms. In this study, we demonstrate that BMP-7 induces MET through Snail1 in rat liver fibrosis cells (post-EMT).

## Materials and methods

### Animals

Adult gender-matched (n=20 each) C57BL rats weighing 200±10.2 g were purchased from Tongji University Laboratories (Shanghai, China) and fed with a commercial diet and water. All animal experiments were performed according to the National Institutes of Health (NIH) guidelines for the ethical care and use of laboratory animals, and the experimental protocol was approved by the Tongji Animal Care and Use Committee of China.

### Rat liver fibrosis models

A total of 40 adult numbered rats were sorted into liver fibrosis model and normal control groups. A total of 20 rats in the liver fibrosis group received intraperitoneal injections of 40% CCl_4_ and olive oil admixture (0.5 ml/100 mg, Sigma-Aldrich, St. Louis, MO, USA) tert as previously described (4). Rats were sacrificed after 8 weeks of treatment.

### Chemicals and materials

Glass slides (75×25 mm^2^) were obtained from Gibco (Carlsbad, CA, USA). (3-Acryloxypropyl) trichlorosilane was purchased from Gelest, Inc. (Morrisville, PA, USA). Streptavidin-conjugated Alexa 546, AlexaFluor 488 anti-mouse IgG, BMP-7 and Snail were obtained from Sigma-Aldrich. Mouse anti-E-cadherin antibody was purchased from BD Biosciences (Franklin Lakes, NJ, USA). Concentrated phosphate-buffered saline (10X PBS) was purchased from Lonza (China). Minimal essential medium (MEM), sodium pyruvate, non-essential amino acids, fetal bovine serum (FBS), Superscript III, RNaseOut (RNase inhibitor) and dNTPs were purchased from Lonza. The 384-well polypropylene microarray plates were obtained from Genetix (China). Goat anti-rat cross-adsorbed albumin antibody was obtained from Sigma-Aldrich. Formalin was purchased from Fisher Scientific (China). ApopTag Red *in situ* Apoptosis Detection kit was obtained from Chemicon (China). DAPI stain mounting media were purchased from Vectorshield (China).

### Cell culture and transfections

Established LEPC cells were obtained from the ATCC collection (LGC Standards-SLU, Barcelona, Spain). Cell lines were maintained in DMEM supplemented with 10% FBS and antibiotics (100 μg/ml ampicillin, 32 *μ*g/ml gentamicin; Sigma-Aldrich). Stable and transient transfections were performed using Lipofectamine reagent (Invitrogen, Carlsbad, CA, USA) according to the manufacturer’s instructions for the generation of stable clones.

### Immunoblot analysis, immunocytochemistry and immuno-precipitation

Tissue and cell lysates were prepared and immunoblot analysis was performed as described previously (10). Band intensity was determined using ImageMaster 2D Elite version 4.01 software (Amersham/GE Healthcare, Uppsala, Sweden). For immunoprecipitation, after liver cells were treated with Snail or BMP-7 for 48 h, the cells were lysed in buffer [50 mM Tris-HCl, pH 8.0, 150 mM NaCl, 5 mM ethylenediaminetetraacetic acid (EDTA) and 0.5% Nonidet P-40 (NP-40)] and centrifuged at 16,000 × g for 15 min to remove debris. Cleared lysates were subjected to immunoprecipitation with antibodies. For immunocytochemistry, cells were fixed in 4% paraformaldehyde at room temperature for 15 min, permeabilized in 5% Triton X-100 for 5 min and then stained using pAbs. The secondary antibodies used were anti-mouse Alexa Fluor 594 dye conjugate and anti-rabbit Alexa Fluor 488 dye conjugate (Molecular Probes/Life Technologies, Carlsbad, CA, USA). Nuclei were stained with 4′,6-diamidino-2-phenylindole (DAPI Blue; Molecular Probes/Life Technologies). After mounting, the cells were visualized using a multiphoton confocal laser-scanning microscope (Carl Zeiss, Thornwood, NY, USA).

### Co-immunoprecipitation and western blot assays

Briefly, LEPC cells were transiently transfected with the indicated vectors for 48 h. Lysates were then obtained in immunoprecipitation buffer (50 mM Tris-HCl, pH 8.0, 150 mM NaCl, 5 mM EDTA, 0.5% NP-40) containing protease and phosphatase inhibitors (2 *μ*g/ml aprotinin, 1 *μ*g/ml leupeptin, 1 mM PMSF, 1 mM Na_3_VO_4_, 10 mM NaF) and precleared with Sepharose G-beads. Supernatants were subjected to overnight incubation with anti-HA affinity matrix (Roche Diagnostics, Indianapolis, IN, USA) or Sepharose G-beads coated with anti-rat IgG as an immunoprecipitation control. Immunoprecipitates were resolved by PAGE on 7.5–12% SDS gels, transferred to membranes and incubated with the indicated antibodies. The membranes were then developed using ECL reagent following the manufacturer’s instructions (Amersham Pharmacia Biotech, Piscataway, NJ, USA). Blots were incubated with rat anti-HA (Roche Diagnostics; 1:100) or mouse anti-flag (Sigma-Aldrich; 13:000). The secondary antibodies used were HRP-coupled goat anti-rat (Pierce Biotechnology, Inc., Rockford, IL, USA; 110:000) or sheep anti-mouse (Pierce Biotechnology, Inc.; 11:000). For detection of E-cadherin, α-smooth muscle actin (α-SMA) and Snail expression, western blotting was performed on whole-cell lysates using rat anti-E-cadherin ECCD2 mAb (1:200, produced in our laboratory from the ECCD2 hybridoma, a gift of M. Takeichi, Ricken Center, Japan), mouse anti-α-SMA (1:500, Dako, Carpinteria, CA, USA) or rat anti-Snail (Roche Diagnostics), followed by HRP-coupled secondary antibodies.

### Real-time quantitative PCR (RT-PCR) analysis

RT-PCR analysis of cDNA samples was performed with specific primers designed using Primer Express software (Applied Biosystems, Foster City, CA, USA). The primers used for Snail were 5′-AAGGATCTCCAGGCTCGAAAG-3′ (forward) and 5′-GCTTCGGATGTGCATCTTGA-3′ (reverse) and those used for β-actin were 5′-GCAAAGACCTGTACGCCAACA-3′ (forward) and 5′-TGCATCCTGTCGGCAATG-3′ (reverse). Total RNA was extracted from cultured cells using an RNeasy kit (Qiagen, Hilden, Germany) according to the manufacturer’s instructions. cDNA was synthesized using 1 *μ*g of RNA with avian myeloblastosis virus reverse transcriptase (Promega, Madison, WI, USA) and oligo(dT) primers. Transcript levels were assessed by RT-PCR (ABI 7300; Applied Biosystems) and all experiments were normalized to β-actin.

### In vivo ubiquitination assay

The cells were treated with 10 *μ*M MG132 for 6 h, 24 h after transfection. The treated cells were then harvested with PBS containing 10 mM *N*-ethylmaleimide (NEM) and 1 mM dithiothreitol (DTT). The cells were washed with PBS, centrifuged and subjected to one freeze-thaw cycle. Cell pellets were then resuspended in 200 *μ*l buffer 1 [10 mM Tris-HCl, pH 7.5, 10 mM NaCl, 0.5% NP-40, 5 mM EDTA, 1 mM ethylene glycol tetraacetic acid (EGTA), 10 mM NEM, 1 mM DTT, 5 mM NaF, 1 mM Na_3_VO_4_ and protease inhibitor cocktail] and sonicated in a water bath (Bioruptor; Diagenode, Denville, NJ, USA). Next, 500 *μ*l buffer 2 (20 mM Tris-HCl, pH 7.5, 0.5 M NaCl, 0.5% NP-40, 5 mM EDTA, 1 mM EGTA, 10 mM NEM, 1 mM DTT, 5 mM NaF, 1 mM Na_3_VO_4_ and protease inhibitor cocktail) was added and the extracts were subjected to a 30-min rotation at 4°C. The extracts were then centrifuged. We added 2 *μ*g of anti-Flag M2 antibody and protein A/G beads to the supernatant, which was then incubated for 2 h. The beads were then washed three times, resuspended in loading buffer and boiled. Immunoblotting was performed as described above.

### Groups for a role for Snail in rat liver fibrosis

A total of 15 adult numbered rats were randomly sorted into 3 groups: i) normal control group: 5 rats received intraperitoneal injections of olive oil (0.5 ml/100 mg) twice per week; ii) liver fibrosis model group: 5 rats received intraperitoneal injections of 40% CCl_4_ and olive oil admixture (0.5 ml/100 mg); iii) BMP-7-treated group: 5 rats received intraperitoneal injections of 40% CCl_4_ and olive oil admixture (0.5 ml/100 mg) twice per week and BMP-7 (300 *μ*g/kg) at same time. Primary rat hepatocytes (1×10^6^/dish) were cultured for 48 h in F12 medium containing 10% fetal bovine serum and 2 *μ*g/ml insulin until they had adhered, which was marked as ‘−’; Primary rat hepatocytes (1×10^6^/dish) were cultured for 48 h in F12 medium containing 10% fetal bovine serum and 2 *μ*g/ml insulin until they had adhered. To induce EMT, the media were replaced with F12 media supplemented with 0.5% fetal bovine serum and 200 mg/*μ*l insulin containing Snail for 96 h, which was marked as ‘+’.

### Statistical analysis

All results shown in the bar graphs are expressed as the fold ratio relative to untreated or control cells. Statistical analysis was performed using SPSS version 17 statistical software (SPSS Inc., Chicago, IL, USA). Student’s t-test was used when comparing two groups. One-way ANOVA was used when comparing multiple groups, followed by Tukey’s post-hoc test. P<0.001 was considered to indicate a statistically significant result.

## Results

### General remarks and groups

None of the animals died during the study period. Body weight gain was lower in the Snail-treated compared to the control rats (data not shown). A total of 40 adult numbered rats were randomly sorted into 4 groups: i) normal control group: 5 rats received intraperitoneal injections of olive oil (0.5 ml/100 mg) twice every week; ii) liver fibrosis model group: 10 rats received intraperitoneal injections of 40% CCl_4_ and olive oil admixture (0.5 ml/100 mg) tert as previously described (4); iii) Snail-treated group: 10 rats received intraperitoneal injections of 40% CCl_4_ and olive oil admixture (0.5 ml/100 mg) twice every week and Snail (500 *μ*g/kg) at the same time; iv) BMP-7-treated group, 10 rats received intraperitoneal injections of 40% CCl_4_ and olive oil admixture (0.5 ml/100 mg) twice every week and BMP-7 (300 *μ*g/kg) at the same time. The rats were sacrificed after 8 weeks of treatment.

### A role for Snail in rat liver fibrosis

**Figure 1:**
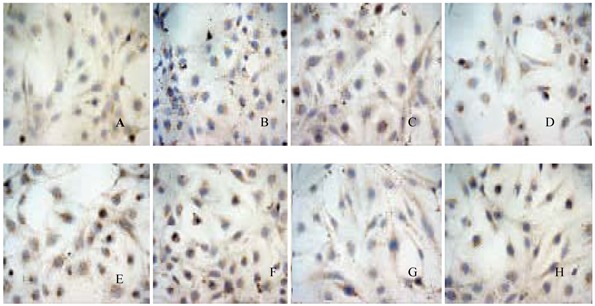
Protein expression of (A–D) E-cadherin and (E–H) α-SMA in rat hepatic tissue (immunofluorescence method). (A and E) Control group; (B and F) Snail-treated group; (C and G) BMP-7-treated group; (D and H) Snail + BMP-7-treated group. α-SMA, α-smooth muscle actin; BMP-7, bone morphogenetic protein-7.

**Figure 2:**
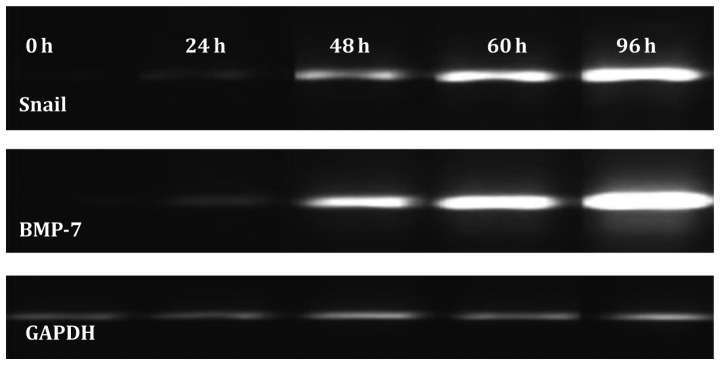
Expression of Snail protein and BMP-7 mRNA in CCl_4_-induced rat liver fibrosis during EMT at different time points (from 0 to 96 h). BMP-7, bone morphogenetic protein-7; EMT, epithelial-mesenchymal transition.

**Figure 3:**
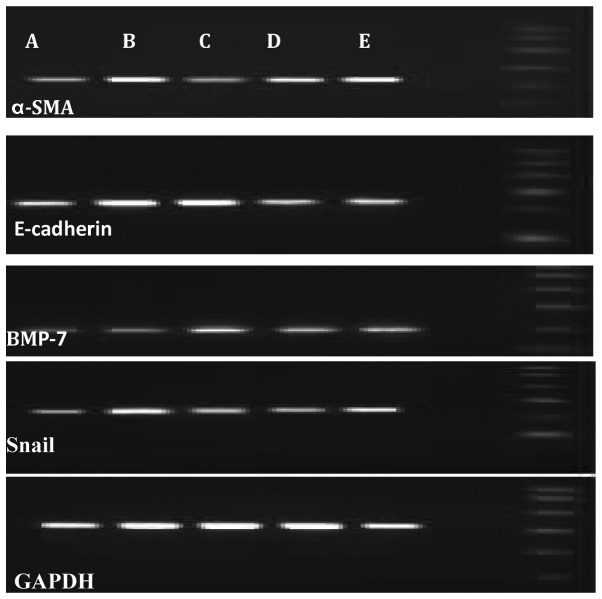
Expression of α-SMA, E-cadherin, BMP-7 and Snail mRNA in CCl_4-_induced rat liver fibrosis during EMT. (A) Normal group; (B) Snail-treated group; (C) BMP-7 (100 ng/ml)-treated group; (D) Snail + BMP-7-treated group; (E) TGFβ1-treated group. α-SMA, α-smooth muscle actin; BMP-7, bone morphogenetic protein-7; EMT, epithelial-mesenchymal transition.

**Figure 4:**
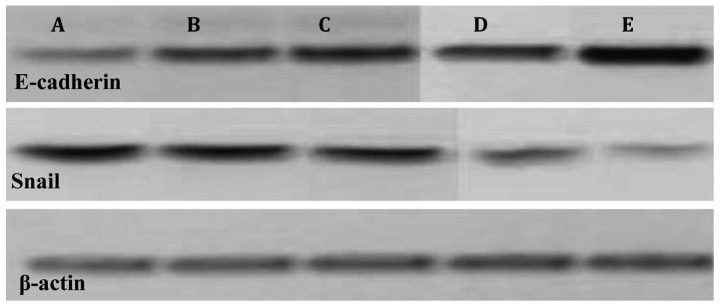
Western blot analysis of the expression of α-SMA and E-cadherin protein in CCl_4_-induced rat liver fibrosis during EMT. (A) TGFβ1-treated group; (B) normal group; (C) Snail-treated group; (D) liver fibrosis model group; (E) BMP-7-treated group. α-SMA, α-smooth muscle actin; EMT, epithelial-mesenchymal transition; BMP-7, bone morphogenetic protein-7.

**Figure 5:**
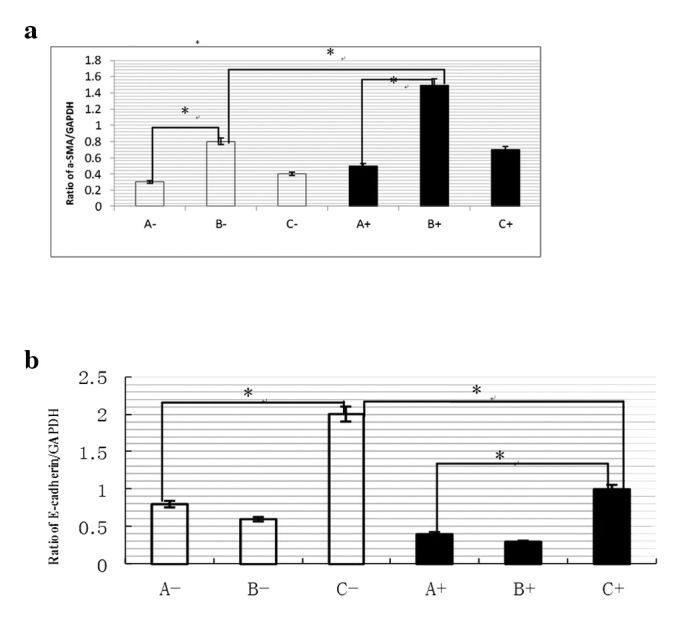
Ratio of α-SMA (a) and E-cadherin/GAPDH (b) in each group. (A) control group; (B) liver fibrosis model group; (C) BMP-7-treated group. −, Primary rat hepatocytes (1×10^6^/dish) were cultured for 48 h in F12 medium containing 10% fetal bovine serum and 2 *μ*g/ml insulin until they had adhered. +, Primary rat hepatocytes (1×10^6^/dish) were cultured for 48 h in F12 medium containing 10% fetal bovine serum and 2 *μ*g/ml insulin until they had adhered. To induce EMT, the media was replaced with F12 media supplemented with 0.5% fetal bovine serum and 200 mg/*μ*l insulin containing Snail for 96 h. ^*^P<0.001. α-SMA, α-smooth muscle actin; BMP-7, bone morphogenetic protein-7; EMT, epithelial-mesenchymal transition.

The present study demonstrated that treatment with Snail induced EMT and increased liver injury during CCl_4_-induced liver fibrosis in rats. This was accompanied by increased expression of liver fibrosis mesenchymal markers, including α-SMA, but inhibition of E-cadherin (P<0.001; Figs. 1–5). The ratio of α-SMA/GAPDH in liver fibrosis model group was higher than that in control group, with 200 mg/*μ*l insulin containing Snail-treated for 96 h, ratio of α-SMA increased significantly in liver fibrosis model group than control group. But this was reverse in ratio of E-cadherin/GAPDH (P<0.001; Fig. 5).

### A role for Snail in rat liver fibrosis

The present study demonstrated that rats treated with Snail had increased hepatic fibrosis in CCl_4_-induced liver injury. This was accompanied by increased expression of hepatic fibrosis mesenchymal markers, including α-SMA, but repression of E-cadherin (Figs. 1–4).

### A role for BMP-7 in rat liver fibrosis

We demonstrated that CCl_4_-induced fibrosis is reversed in rats treated with BMP-7. Significantly more BMP-7 and less Snail mRNA were expressed in the hepatic fibrosis model group than in the controls (P<0.001; Figs. 1–4). This was accompanied by reduced expression of hepatic fibrosis mesenchymal markers, including α-SMA, but increased expression of E-cadherin (Fig. 1). Thus, a strategy that specifically increased BMP-7 in myofibroblasts from cirrhotic livers tended to reverse the myofibroblastic phenotype and caused the cells to acquire a more quiescent and epithelial phenotype.

## Discussion

BMP-7 induces MET through Snail1, which represses α-SMA by binding to E-box promoter elements (11). In the present study, Snail stimulation of epithelial liver fibrosis cells resulted in a mesenchymal phenotype with fibroblastoid appearance and loss of E-cadherin. However, the underlying mechanism has not yet been elucidated. Based on our results, we hypothesize that these liver fibrosis characteristics are Snail-dependent. Inhibition of Snail1 causes the downregulation of Nanog and CD_44_ and loss of self-renewal, as evidenced by decreased liver fibrosis formation. Liver fibrosis cells are more mesenchymal in character, with increased Snail1, Zeb1 and Zeb2 mRNA expression and decreased E-cadherin expression. Notably, although Smads and Snail proteins are known to play a central role in liver fibrosis cell growth, Notch signaling is also capable of inhibiting liver fibrosis growth through the induction of EMT (12). Notch1 is known to regulate Snail and Slug mRNA levels, but efforts have not been made to examine alternative functions of NICD and Snail expression in liver fibrosis (13). In addition, Notch1 is involved in the mesenchymal program by activating Snail expression in liver fibrosis development (10). However, since Notch signals and cellular functions vary according to cell type and cellular environment, these inconsistencies may be caused by the different cell types and conditions. We hypothesized that ROS stress upregulated Snail mRNA and protein expression (14). E-cadherin expression decreased in Snail-overexpressed cells compared with control cells (P<0.001). Our study provides one clue for understanding the complex regulation mechanism of p53, MDM2, Notch1 and Snail in the EMT process. The regulation of these proteins and their physiological contribution to EMT require further investigation. However, the mechanism that we have described presents substantial evidence of cross-interference between the Notch and Snail signaling pathways, which may be mediated by BMP-7. In addition, Snail1 is one of a number of regulators of EMT, and thus manipulation of multiple factors may be required to fully inhibit liver fibrosis initiation.
